# The adult murine heart has a sparse, phagocytically active macrophage population that expands through monocyte recruitment and adopts an ‘M2’ phenotype in response to Th2 immunologic challenge

**DOI:** 10.1016/j.imbio.2015.01.013

**Published:** 2015-07

**Authors:** Katie J. Mylonas, Stephen J. Jenkins, Raphael F.P. Castellan, Dominik Ruckerl, Kieran McGregor, Alexander T. Phythian-Adams, James P. Hewitson, Sharon M. Campbell, Andrew S. MacDonald, Judith E. Allen, Gillian A. Gray

**Affiliations:** a BHF/University Centre for Cardiovascular Science, Queen's Medical Research Institute (QMRI), University of Edinburgh, 47 Little France Crescent, Edinburgh EH16 4TJ, Scotland, United Kingdom; b Centre for Inflammation Research, QMRI, University of Edinburgh, 47 Little France Crescent, Edinburgh EH16 4TJ, Scotland, United Kingdom; c Institute of Immunology and Infection Research (IIIR), The King's Buildings, University of Edinburgh, Charlotte Auerbach Road, Edinburgh EH9 3FL, Scotland, United Kingdom; d Manchester Collaborative Centre for Inflammation Research (MCCIR), University of Manchester, Grafton Street, Manchester M13 9NT, England, United Kingdom

**Keywords:** Mϕ, macrophages, cMϕ, cardiac macrophages, lMϕ, liver macrophages, pMϕ, peritoneal macrophages, IL, interleukin, TNF, tumour necrosis factor, Th2, T helper 2, CSF-1R, macrophage colony stimulating factor 1 receptor, CCR2, C-Cchemokine receptor type 2, RV, right ventricle, LV, left ventricle, Macrophage, Heart, Helminth infection

## Abstract

Tissue resident macrophages have vital homeostatic roles in many tissues but their roles are less well defined in the heart. The present study aimed to identify the density, polarisation status and distribution of macrophages in the healthy murine heart and to investigate their ability to respond to immune challenge. Histological analysis of hearts from CSF-1 receptor (*csf1-GFP*; MacGreen) and CX_3_CR1 (*Cx3cr1*^*GFP/+*^**)** reporter mice revealed a sparse population of GFP positive macrophages that were evenly distributed throughout the left and right ventricular free walls and septum. F4/80+CD11b+ cardiac macrophages, sorted from myocardial homogenates, were able to phagocytose fluorescent beads *in vitro* and expressed markers typical of both ‘M1’ (IL-1β, TNF and CCR2) and ‘M2’ activation (Ym1, Arg 1, RELMα and IL-10), suggesting no specific polarisation in healthy myocardium. Exposure to Th2 challenge by infection of mice with helminth parasites *Schistosoma mansoni*, or *Heligmosomoides polygyrus*, resulted in an increase in cardiac macrophage density, adoption of a stellate morphology and increased expression of Ym1, RELMα and CD206 (mannose receptor), indicative of ‘M2’ polarisation. This was dependent on recruitment of Ly6ChighCCR2+ monocytes and was accompanied by an increase in collagen content.

In conclusion, in the healthy heart resident macrophages are relatively sparse and have a phagocytic role. Following Th2 challenge this population expands due to monocyte recruitment and adopts an ‘M2’ phenotype associated with increased tissue fibrosis.

## Introduction

Well characterised resident macrophage (Mϕ) populations exist in the liver (as Kupffer cells), in the brain (microglia), in the lung (alveolar Mϕ), as well as the peritoneal cavity and bone marrow. In the heart there is plentiful evidence for robust monocyte recruitment after surgically induced myocardial injury (Frangogiannis 2006; Nahrendorf et al. 2007; McSweeney et al. 2010). However, until recently (Pinto et al. 2012; Epelman et al. 2014a), there had been relatively little focus on the regulation, activation status and function of the resident cardiac Mϕ population. This is important, as resident cells in the heart are likely to play a key role in determining the immediate response to ‘sterile’ injury or immunologic challenge (Epelman et al. 2014b).

It is clear that resident Mϕ populations are heterogeneous in terms of origin, activation status and function, due in part to age and gender but also to stimuli present in their local environment (Mosser and Edwards 2008; Davies et al. 2013). Recent studies have suggested that resident cardiac Mϕ (cMϕ) are derived in part from the embryonic yolk sac and liver (Epelman et al. 2014a) but are also replenished from the circulating monocyte population (Molawi et al. 2014). While some studies suggest that they are an abundant population (Pinto et al. 2012; Heidt et al. 2014), others report that they are relatively sparse in the healthy myocardium (Frangogiannis et al. 2002; Macri et al. 2012; Yan et al. 2013). The reasons for these discrepancies are unknown but differences in detection methods e.g. specificity of antibodies in immunohistochemistry and flow cytometry, or use of reporter mice, may have contributed to these varying outcomes.

Resident Mϕ perform varying roles, including maintenance of tissue homeostasis by scavenging and phagocytosing of apoptotic cells arising during normal cell turnover (Lech et al. 2012; Davies et al. 2013). They also act as sentinels of damage and infection, performing immune effector functions. For this reason, Mϕ are highly plastic and signals that they encounter within tissue can alter their activation state, number and function (Mosser and Edwards 2008; Mylonas et al. 2009). Thus both sterile injury and bacterial infection can stimulate them to adopt a ‘classical’ or ‘M1’ phenotype that instigates further inflammatory cell recruitment through the production of pro-inflammatory mediators, such as IL-1β and TNF (Gordon and Taylor 2005; Mosser and Edwards 2008). In contrast, phagocytosis of apoptotic cells (Voll et al. 1997; McDonald et al. 1999) or exposure to Th2 type cytokines, such as IL-4, IL-13 and IL-10 can lead to adoption of an ‘alternatively-activated’ or ‘M2’ Mϕ phenotype. These ‘M2’ Mϕ tend to and typically express markers such as Ym1 and RELMα (Nair et al. 2005), suppress inflammation (Mosser and Edwards 2008; Gordon and Martinez 2010) and promote tissue repair, but also fibrosis (Mantovani et al. 2013; Hofmann et al. 2014). The abundant resident cardiac population described by Pinto et al. (2012) was polarised towards an alternatively activated or ‘M2’ phenotype and also stellate shaped but the underlying mechanism was not investigated. Th2 conditions, such as those arising during helminth infection, promote alternative macrophage activation (Gordon and Martinez 2010; Allen and Wynn 2011), and can also cause expansion of the tissue Mϕ population by resident cell proliferation, rather by recruitment of monocytes from the circulation (Jenkins et al. 2011). The response of the heart to this challenge has not however been investigated.

Our own immunohistochemical studies of Mϕ recruitment following myocardial injury (McSweeney et al. 2010) support suggestions that the resident population of the healthy heart is relatively sparse rather than abundant. The present study was designed to clarify the status of the resident cMϕ population more accurately in terms of density, localisation, function and polarisation status using 2 different Mϕ reporter mouse lines, CSF-1 receptor (*csf1-GFP*; MacGreen; Sasmono et al. 2003) and CX_3_CR1 (*Cx3cr1*^*GFP/+*^; Jung et al. 2000) reporter mice, as well as flow cytometry and i*n vitro* functional analysis. The study also sought to determine the response of cMϕ to Th2 type immunological challenge (MacDonald et al. 2002) following infection with 2 separate helminth parasites *Schistosoma mansoni* (*S. mansoni*) or *Heligmosomoides polygyrus* (*H. polygyrus*).

## Materials and methods

### Mice

Mice (8–12 weeks old) were bred and maintained in conventional barrier unit facilities at the University of Edinburgh. These units are regularly tested in accordance with the Felasa 2014 recommendations, which involves testing for various infectious agents, including parasites. Experimental mice were age and sex matched, and C57BL/6 unless otherwise stated.

### Ethics statement

All animal work was compliant with IACUC guidelines, conducted in accordance with the UK Government Animals (Scientific Procedures) Act 1986 and was approved by the University of Edinburgh Ethical Review Committee.

### Transgenic mice and helminth infection

Naïve hearts from MacGreen mice, in which cells expressing the c-*fms* gene (CSF-1 receptor; i.e. Mϕ) are positive for enhanced green fluorescent protein (EGFP) (Sasmono et al. 2003), were kindly donated by Prof David Hume's lab (Roslin Institute, University of Edinburgh). In addition, hearts were collected from *Schistosoma mansoni* (*S. mansoni*) infected *Cx3cr1*^*GFP/+*^ mice. In these *Cx3cr1*^*GFP/+*^ mice one allele of the CX_3_CR1 gene has been replaced by the gene encoding GFP (Jung et al. 2000). Hearts were also obtained from *Heligmosomoides polygyrus* (*H. polygyrus*) infected *Cx3cr1*^*GFP/+*^ mice and from mice lacking CCR2 (CCR2KO; Boring et al. 1997). The infections were carried out according to published protocols. Briefly, *Cx3cr1*^*GFP/+*^ mice were infected either percutaneously with ∼80 *S. mansoni* cercariae and hearts recovered 8 weeks later (Phythian-Adams et al. 2010), or with 200 *H. polygyrus* L3 by oral gavage before recovery of hearts 28 days later (Hewitson et al. 2011). These time points were chosen because they reflect the chronic infection stage, with egg production by adults, for these two distinct parasites.

### Immunohistochemistry (IHC)

Frozen sections from MacGreen hearts (fixed in 4% paraformaldehyde and snap frozen), were air-dried for 20 min before mounting and coverslipping with Fluoromount aqueous mounting medium (Sigma). GFP positive Mϕ were visualised using the Zeiss Axioskop 2mot+ or Confocal LSM710.

As *Cx3cr1*^*GFP/+*^ heart sections were fixed in 10% formalin following collection, dampening the GFP signal, GFP was detected in these hearts using a specific antibody (rabbit anti-GFP; 1:1000; AbCam Ab290), rather than direct detection of the GFP signal. Expression of GFP and of the ‘M2’ markers Ym-1 (rabbit anti-Ym1; 1/100; StemCell Technologies) and RELMα (rabbit anti-RELMα; 0.25 μg/mL; Peprotech) was assessed in heart sections by indirect immunoperoxidase staining.

Briefly, the paraffin embedded tissue sections were deparaffinised and rehydrated. After high temperature antigen unmasking (citrate buffer), endogenous peroxidase was quenched with aqueous 2–3% H_2_O_2_ (Sigma–Aldrich, UK) for 15–20 min. For GFP staining, slides were then incubated for 30 min in 2.5% Horse Serum (IMPRESS kit, Vector Labs), followed by incubation with primary antibody for 1 h at room temperature. For Ym1 and RELMα, sections were blocked in goat serum buffer (10% goat serum in phosphate buffered saline; PBS), then incubated overnight at 4 °C with primary antibodies. The secondary antibodies were goat anti-rabbit biotin for RELMα and Ym1 (1 mg/ml, Dako Cytomation, Denmark) and anti-rabbit IMPRESS reagent for GFP (IMPRESS kit, Vector Labs). Peroxidase-labelled ABC reagent for Ym1 and RELMα and DAB substrate (Vector Laboratories, UK) for all three were then added for signal visualisation. Finally, the sections were counterstained with haematoxylin, dehydrated through ethanol and xylene and mounted with DPX mountant (Sigma).

To carry out picro-sirius red (PSR) staining for collagen, sections were deparaffinised and rehydrated as above, before treatment in haematoxylin for 8 min. After washing, these were then stained in picro-sirius red for 1 h, before washing in acidified water and dehydration and mounting.

For quantification, sections were tiled at 40× magnification and fields were randomly selected in the heart (Image Pro6.2, Stereologer Analyser 6 MediaCybernetics). Hearts were divided into specific areas (i.e. left/right ventricular free walls, septum and sub-regions) for GFP+ macrophage distribution quantification (% area stained). For quantification of Ym-1, a secreted protein, and PSR the % area stained was calculated in randomly assigned fields. Individual GFP and RELMα stained cells were counted in 5 random fields. This technique was also applied to quantification of “stellate” cells (cells with protruding dendrites).

### Peritoneal exudate cell (PEC) extraction

The PECs were harvested by thorough washing of the peritoneal cavity of euthanised naïve WT mice with 5 ml of ice-cold PBS.

### Digestion of hearts and livers to obtain single cell suspensions

Hearts and livers from naïve mice (WT, MacGreen or *Cx3cr1*^*GFP/+*^) or those infected with *H. polygyrus* (*Cx3cr1*^*GFP/+*^) were digested to obtain single cell suspensions for flow cytometric analysis. Firstly, perfusion was carried out through the abdominal aorta with heparinised Krebs solution (10 IU/ml heparin) in order to remove blood monocytes. Where perfusion was not possible, mice received an i.v. injection of CD45 FITC antibody (1 μg in 5 USP units heparin in 100 μl PBS; Invitrogen) 2–10 min before cull in order to stain for blood leukocytes and to allow them to be distinguished from tissue Mϕ, as previously described (Tagliani et al. 2011). Tissue was then harvested by dissection and chopped into small pieces. These were digested in Collagenase II and DNase 1 (600 U/ml Collagenase II; CLS-2 Worthington, 60 U/ml DNAse 1; Ambion, Warrington) in Hank's Balanced Salt Solution (HBSS; GIBCO) at 37 °C for 30 min following dissociation by GentleMacs dissociator (Miltenyl). The digested tissue was gently pressed through a 70-μm cell strainer using a flattened pestle and the cell strainer was then washed with PBS (GIBCO). The cells were centrifuged at 300 × *g* for 5 min and then washed once again in PBS.

### Flow cytometry and fluorescence associated cell sorting (FACS)

0.5–3 × 10^6^ singly suspended cells (from heart and liver digests, or peritoneal lavage) or 60 μl blood (extracted from the tail vein into 3.2% citrate buffer) were placed in FACS tubes (BD). All samples except the blood were then blocked with 10% mouse serum for 20 min on ice. This was followed by staining of all samples for 30 min on ice with the antibodies of interest at the appropriate dilution (in PBS, 10% mouse serum) as determined by titration. The antibodies were directly fluorochrome conjugated and anti-mouse. The antibodies included anti-F4/80 PE, anti-CD45 PE Cy5, anti-CD45.2 PE Cy7, anti-CD11b-Alexafluor 700, Ly6C PerCp cy5.5, CD206 APC, Ly6G PE Cy7 and Ly6G Pacific Blue (all from Biolegend). Cells were then washed in PBS before acquisition and analysis (BD FACS LSR Fortessa and FlowJo software).

Cells passed through the cytometer were gated by granularity and size, and singlets selected to exclude cell clumps. Dead cells (DAPI+), and any CD45 FITC+ leukocytes labelled prior to cull, were excluded from analysis of tissue Mϕ. CD45+ CD11b+ F4/80+ Ly6G−, and in the case of MacGreen animals, GFP+ Mϕ were selected. A thorough gating strategy is shown in Fig. 2. Confirmatory experiments in CSF-1R GFP (MacGreen) mice show that the F4/80+CD11b+ cMϕ are also GFP+ (Supplementary Fig. 1), and are therefore cMϕ. For FACS of Mϕ from naïve WT and MacGreen mice, sorting was carried out using the FACsAria II. Mϕ were frozen at −80 °C in TRIizol Reagent (Invitrogen) before RNA extraction and real-time RT-PCR. 12–15,000 Mϕ were recovered from whole naïve hearts and >100,000 were collected from PEC and liver.

### RNA extraction and real-time RT PCR

RNA was extracted from the above purified WT cardiac, liver and peritoneal Mϕ using the Direct-zol™ RNA MiniPrep kit (Cambridge Bioscience), or TRIZOL method, according to the manufacturers instructions and this was then reverse transcribed to cDNA (Applied Biosystems high capacity cDNA reverse transcription kit). TAQman^®^ gene expression assays were used to detect GAPDH (Mm99999915_gl gapdh), Ym1 (Mm00657889_mM Chi313), Arginase 1 (Mm00475988_ml Arg1), Interleukin (IL-) 10 (Mm00439614_ml Il10), Tumour Necrosis Factor (Mm00443260_gl Tnf), IL-1β (Mn00434228_m1 il1b), RELMα (Mm00445109_ml Retnla) and CCR2 (Mm0121-6173_ml Ccr2). mRNA expression levels were normalised for GAPDH expression and presented as fold increases over average cMϕ level analysed in parallel.

### Phagocytosis assay

Mϕ from naïve WT mice were collected by FAC Sorting from the heart and peritoneal cavity as described above. The Mϕ were washed once in DMEM/F-12 (2 mM l-glutamine, 0.25 U/ml penicillin and 100 mg/ml streptomycin; Gibco) and then plated at 12,000–15,000 cells per well in 500 μl of the above medium in 48 well plates. They were allowed to adhere without foetal calf serum (FCS) for 1 h at 37 °C before 250 μl of the medium was removed and replaced with 250 μl of the same medium this time containing 20% FCS to aid phagocytosis, as well as 120,000–150,000 3 μm Fluoresbrite^®^ YG Microspheres (Polysciences, Inc.). As a control, an extra subset of pMϕ was also given 250 μl medium without spheres. These were then placed again at 37 °C for 30 min to allow the Mϕ to phagocytose the microspheres. After this time, the plate was placed on ice, the wells washed once with PBS and then detachment buffer (1L PBS, 0.18 g Glucose, 6 ml EDTA; 0.5 M) added for 15 min (still on ice). The cells were then lifted with a cell scraper and transferred to chilled FACS tubes where they were washed once in PBS and then fixed in 5% formalin before acquisition and analysis (BD FACsCalibur and FlowJo software). Mϕ were gated by size (SSC) and those that had phagocytosed spheres visualised in the FITC channel. From this, % phagocytosis of spheres by Mϕ was calculated.

### Statistical analysis

All values are expressed as mean ± SEM. Unpaired Student's *t*-test or ANOVA were used for analysis. *p*-values <0.05 denote statistical significance, *p < 0.05, **p < 0.01, ***p < 0.005.

## Results

### The adult murine heart contains a sparse resident Mϕ population that is evenly distributed

**Fig. 1:**
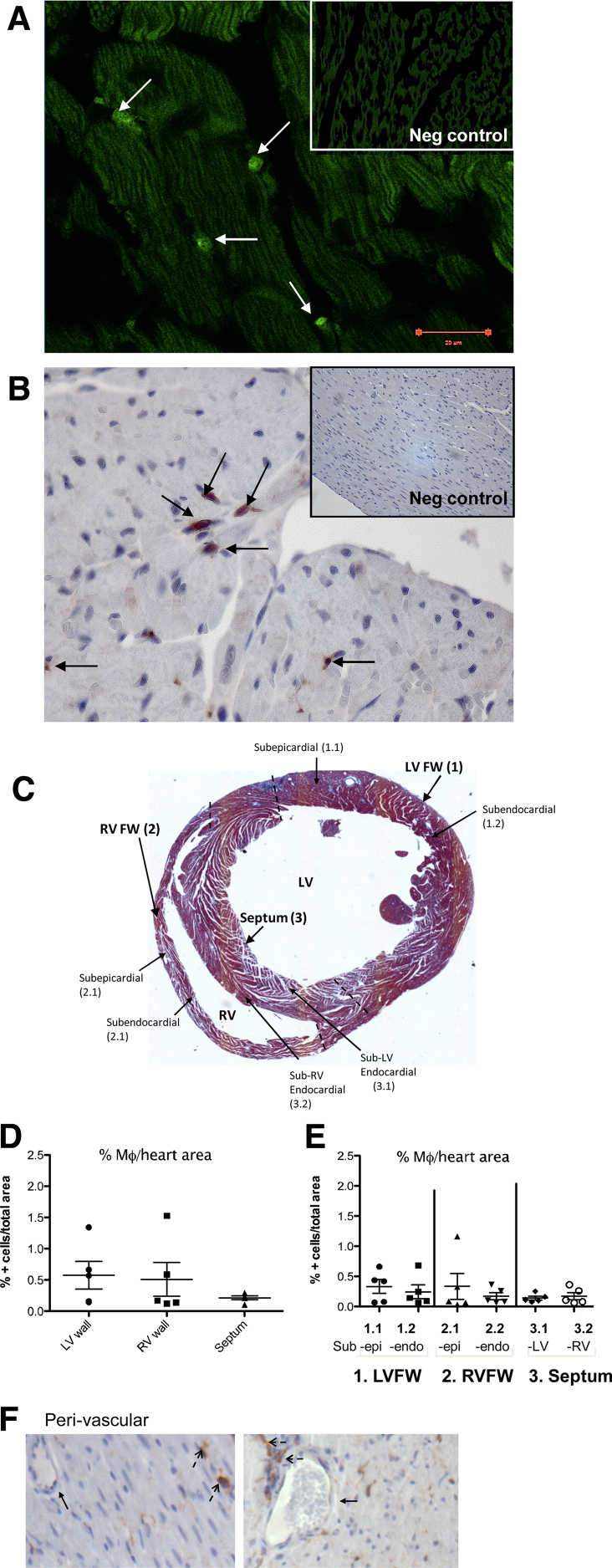
The adult murine heart contains resident CSF-1R+ and CX_3_CR1+ Mϕ that are evenly distributed throughout. (A) Representative micrograph of MacGreen CSF-1R GFP reporter mouse heart section (400× magnification). White arrows indicate representative GFP+ cells. (B) *Cx3cr1^GFP/+^* heart section that has undergone anti-GFP immunohistochemistry (IHC) showing positive cells (black arrows indicate representative GFP stained cells; 400× magnification). Inlaid negative controls are shown at 200× magnification. Hearts sections from *Cx3cr1^GFP/+^* mice were divided into the following subsections as outlined in the schematic transverse heart section (C). Left Ventricular Free Wall (LV FW; 1), Right Ventricular FW (RV FW; 2) and Septum (3), and subsections therein, i.e. subepicardial (outer) areas of LV and RV FWs (1.1 and 2.1; sub-epi), and subendcocardial (inner) areas of LV and RV FWs (1.2 and 2.2; sub-endo). The septum was divided into sub-LV-endocardial (3.1) and sub-RV-endocardial (3.2) sections, those being the areas closer to the LV and RV respectively (C). GFP+ cells were quantified in the LV, RV and septum (D) and the subsections of each (E). Representative micrographs showing perivascular areas (f; black arrows pointing to vessels, dashed arrows to Mϕ).

Histological analysis of hearts from MacGreen CSF-1R GFP reporter mice revealed a sparse population of GFP+ cells (400× magnification; Fig. 1a), with a mostly rounded shape. Flow cytometric evaluation of these GFP+ cells in the heart showed that they were F4/80 + CD11b+ cMϕ (Supplementary Fig. 1a and b). A similar density of GFP+ cells was seen in hearts from *Cx3cr1*^*GFP/+*^ mice (400× magnification; Fig. 1b). Investigation of distribution (Fig. 1c–f) showed that cMϕ density was similar in the right and left ventricular walls (RV and LV respectively) and in the septum (Fig. 1d), although there did seem to be more variation within the ventricles. There was also no particular association with endocardial or epicardial regions of the ventricles or subsections of the septum (Fig. 1e). *Cx3cr1*^*GFP/+*^ cells were found in association with blood vessels, but were not limited to perivascular tissue (Fig. 1f).

### Murine cardiac macrophages (cMϕ) have phagocytic capacity and express both ‘M1’ and ‘M2’ markers

**Fig. 2:**
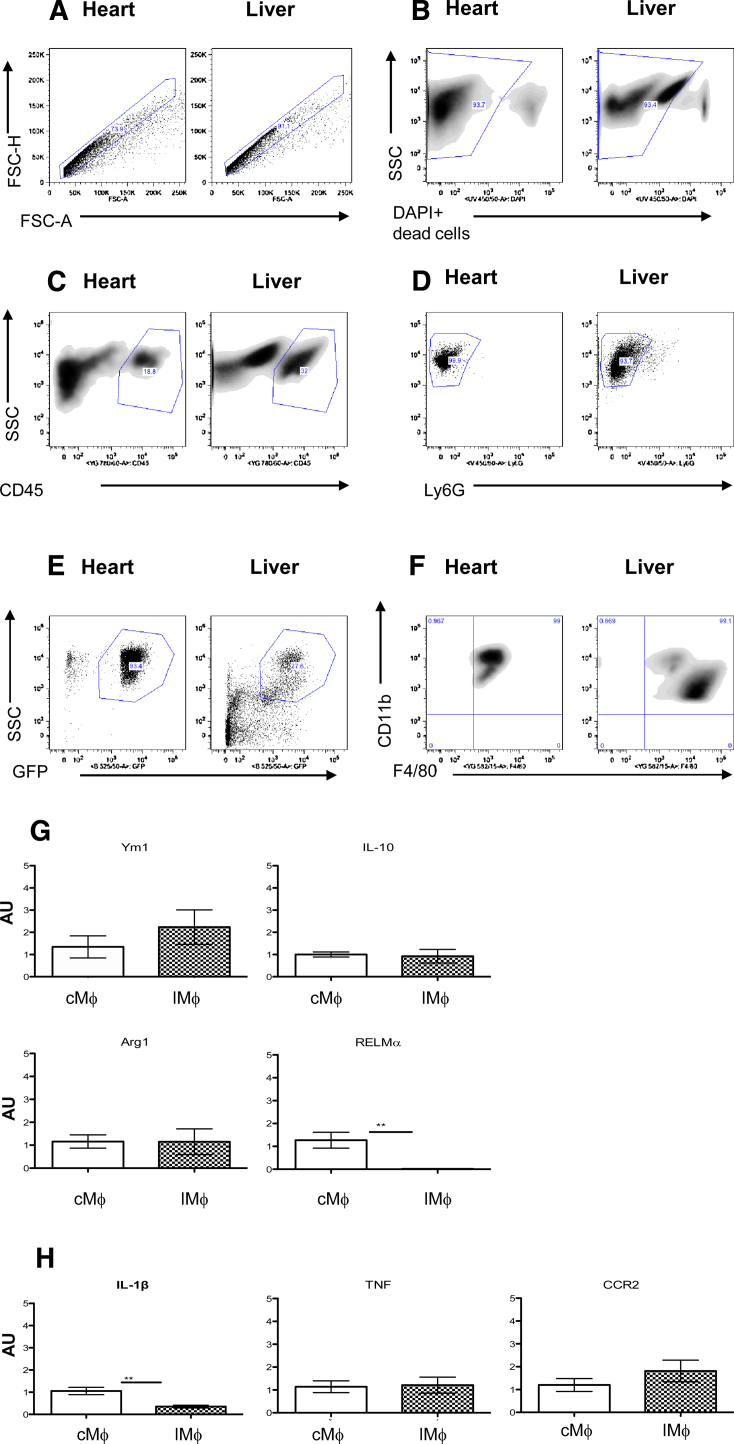
cMϕ are not overtly polarised. (A–F) Gating strategy for FACS of resident heart compared to resident liver Mϕ. Single cell suspensions from MacGreen hearts and livers were gated by granularity and size (by SSC, Side Scatter and FSC, forward scatter) and singlets (A) selected to exclude cell clumps (FSC- H/FSC-A; -height/-area). Dead cells (B) are excluded. CD45+ (C) and Ly6G negative cells are gated on to select leukocytes and to exclude neutrophils (D). Mϕ selected are GFP+ (CSF-1R+; E), CD11b+ and F4/80+ (F). (G, H) Realtime PCR analysis of cMϕ vs LMϕ. Comparison of M2-like Mϕ markers (G; Ym-1, IL-10, Arg1 and RELMα) and M1-like/inflammatory markers (H; iNOS, TNF and CCR2) by realtime PCR. in FACS sorted cMϕ (open columns) vs LMϕ (hashed columns, *n* = 6/7; *p < 0.05, ***p < 0.005; AU = arbitrary units, normalised to GAPDH).

12,000–25,000 F4/80+CD11b+ cMϕ were recoverable by FACS (Fig. 2a–f) from enzymatically digested mouse hearts, representing approximately 200–250 macrophages per mg heart tissue (or ∼0.5–1 cMϕ per mm^2^). cMϕ collected in this way were able to efficiently phagocytose fluorescent microbeads (Supplementary Fig. 2i).cMϕ were then compared to Mϕ from the liver (liver Mϕ; lMϕ), that had undergone a similar digestion protocol to the heart, and from the peritoneal cavity (peritoneal Mϕ; pMϕ), these representing other resident Mϕ populations. Over 100,000 Mϕ were collected from enzymatically digested liver and from peritoneal exudate cells (PEC) using similar gating strategies (Fig. 2a–f and Supplementary Fig. 2a–f). All sorted cMϕ, lMϕ and pMϕ expressed markers typical of both ‘M1’ (IL-1β, TNF, CCR2) and ‘M2’ activation (Ym1, Arg1, RELMα and IL-10). In comparison to the pMϕ population, cMϕ expressed higher levels of all ‘M1’ markers (Supplementary Fig. 2h) and also more IL-1β than lMϕ (Fig. 2h). cMϕ expressed more of the ‘M2’ associated RELMα than lMϕ (Fig. 2g) and more YM1 than pMϕ (Supplementary Fig. 2g). Expression of the other ‘M2’ markers was not significantly increased in cMϕ compared to the other resident Mϕ populations (lMϕ nor pMϕ; Fig. 2g and Supplementary Fig. 2g). However, most transcripts came up at relatively late cycles of the PCR reaction (>30 CT) compared to the housekeeping control gene (GAPDH; mean CT 26) implying that low expression of these markers in Mϕ populations from all 3 sites.

### Th2 challenge is associated with increased numbers of ‘M2’ cMϕ and tissue development of tissue fibrosis

To determine the potential for cMϕ to respond to Th2 immunological challenge, hearts were collected from mice exposed to infection with 2 distinct helminths, the tissue resident trematode *S. mansoni* or the gut-dwelling nematode, *H. polygyrus.*

**Fig. 3:**
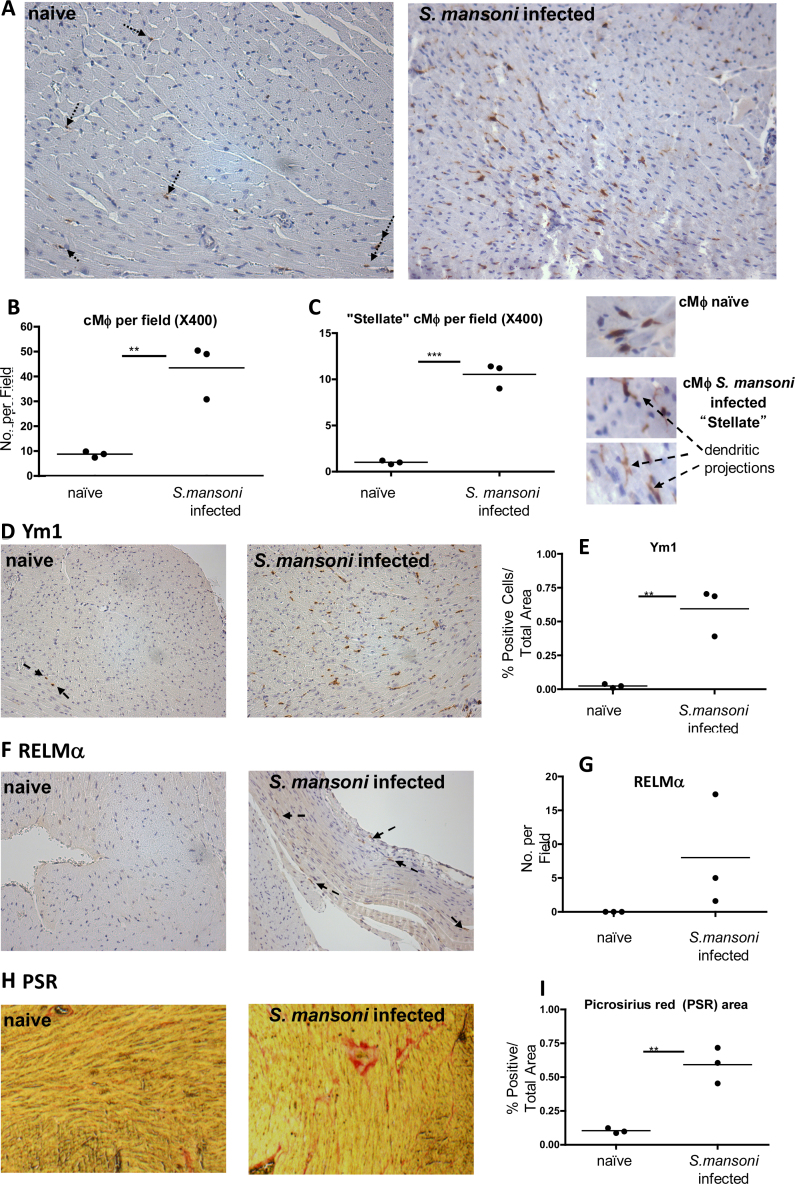
*Schistosoma mansoni* infection changes the frequency, morphology and phenotype of heart resident Mϕ, and increases the collagen content of the heart. (A) Representative *Cx3cr1^GFP/+^* heart sections from naïve and *S. mansoni* infected mice that have undergone anti-GFP IHC showing positive cells (black arrows indicate representative GFP stained cells in naïve mouse hearts. GFP positive cells are clear in infected section; 200× magnification). Number of cMϕ (B) and “stellate” Mϕ (C left) per field in naïve vs *S. mansoni* infected mice. Representative micrographs comparing stellate Mϕ (with dendritic projections) vs “typical” cMϕ (C right). (D) Representative heart sections from naïve and *S. mansoni* infected mice that have undergone Ym1 IHC showing positive cells (black arrows indicate representative GFP staining in naïve hearts; 200× magnification). (E) Quantification of Ym1+ cells per area in naïve vs *S. mansoni* infected heart sections. (F) RELMα+ cells in naïve (left) vs *S. mansoni* infected (right) mouse heart sections (black arrows indicate representative GFP stained cells; 200× magnification). (G) Quantification of RELMα staining in naïve vs *S. mansoni* infected heart sections (statistical analysis could not be carried out as no RELMα was detectable in naïve hearts). (H) Picrosirius Red staining indicating collagen content in naïve vs *S. mansoni* infected heart sections and quantification of % positive area (I). **p < 0.01, ***p < 0.005.

Firstly, in hearts isolated from *S. mansoni* infected *Cx3cr1*^*GFP/+*^ mice, cells immunopositive for GFP were present in significantly greater numbers than in uninfected *Cx3cr1*^*GFP/+*^ mice (Fig. 3a and b; *p* < 0.01). The distribution of GFP+ cells increased uniformly throughout in the hearts of infected animals (data not shown). Infection also resulted in acquisition of a “stellate” shape by GFP+ cells (Fig. 3c, *p* < 0.005). While very few cells in naïve hearts displayed Ym1 immunoreactivity (Fig. 3d left panel), this was significantly increased in the hearts of mice infected with *S. mansoni* (Fig. 3d and e, *p* < 0.01). The presence of alternatively activated Mϕ was confirmed by the presence of RELMα immunopositive cells in hearts from *S. mansoni* infected hearts only (Fig. 3f and g). This was accompanied by an increase in tissue collagen content (Fig. 3h and i).

**Fig. 4:**
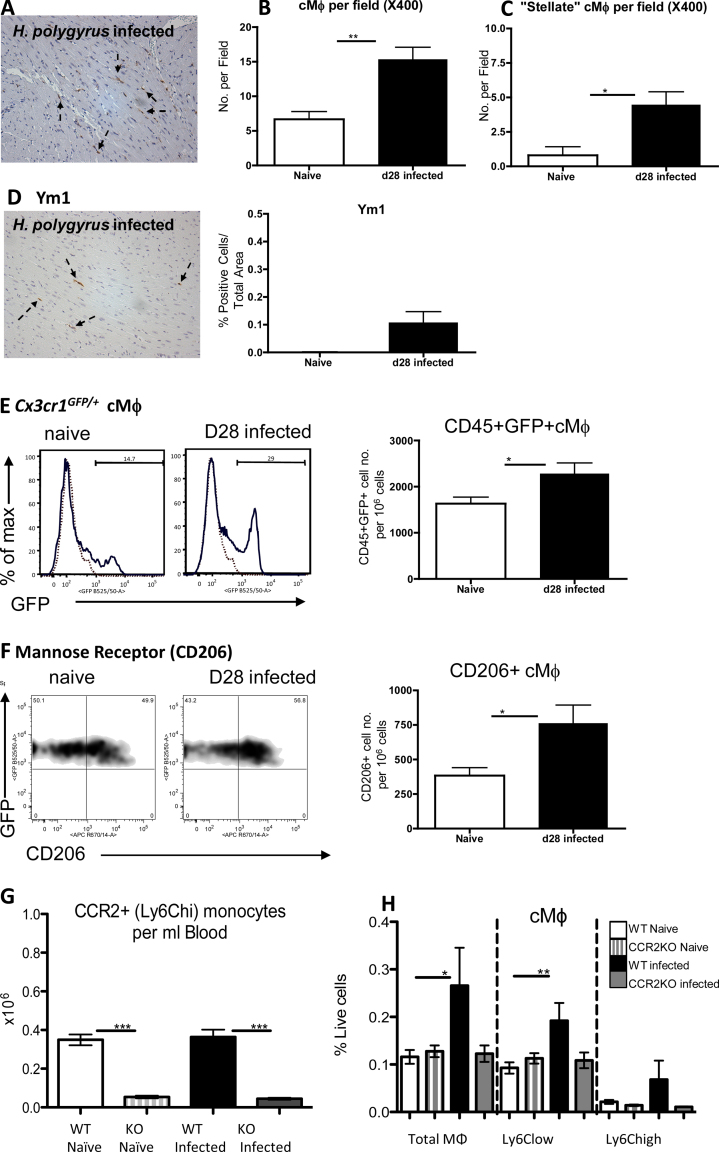
The presence of a *H. polygyrus* infection increases the frequency of heart resident Mϕ and this is largely dependent on monocyte recruitment. (A) Representative micrograph of GFP IHC in heart section from naïve and infected *Cx3cr1^GFP/+^* mice. (B) Number of cMϕ (GFP+) and “stellate” cMϕ (C) per field in *Cx3cr1^GFP/+^* in naïve (open columns) vs mice that had been infected for 28 days with the GI helminth *H. polygyrus* (filled columns). (D) Representative micrograph of Ym1 staining of a d28 infected heart (left panel) and quantification of Ym1+ cells per area in naïve vs 28 days post-infected mice (right panel). (E) Representative histograms of CD45+ GFP+ cells in the hearts of naïve and infected mice (left panel; dotted lines represent GFP-control), with quantification of positive cells acquired by flow cytometry (right). (F) Representative scatter profiles of mannose receptor (CD206) expressing cMϕ (left and middle panel) and positive cell numbers (right panel) in naïve vs d28 *H. polygyrus* infected hearts quantified by flow cytometry. (G) Quantification of Ly6Chigh (CCR2+) monocytes in the blood of WT naïve (open column), CCR2KO naïve (grey and white striped column) mice, and WT (black filled column) and CCR2KO (grey filled column) animals that have been infected for 28 days with *H. polygyrus*. (H) Quantification of cMϕ (total Mϕ-left, Ly6Clow-middle and Ly6Chigh-right) acquired by flow cytometry in naïve WT and CCR2KO mice or those that have been infected with *H. polygyrus*. *p < 0.05, **p < 0.01, ***p < 0.005.

Hearts from *H. polygyrus* infected *Cx3cr1*^*GFP/+*^ mice also displayed greater numbers of GFP positive cells (*p* < 0.01), and were also of a more stellate nature (*p* < 0.05), following infection (Fig. 4a–c), with a trend towards increased Ym1 (Fig. 4d). In a follow on experiment, these hearts were digested for flow cytometry analysis. 10^6^ cells from the *Cx3cr1*^*GFP/+*^ hearts were acquired and the number of GFP+ cMϕ was found to be significantly increased (Fig. 4e; *p* < 0.05), as well as those expressing the ‘M2’ marker mannose receptor (CD206) in infected animals (Fig. 4f right panel; *p* < 0.05).

### Recruitment of CCR2^+^ monocytes contributes to expansion of cMϕ following *H. polygyrus* infection

To determine whether monocyte recruitment contributes to the Mϕ expansion in the heart following infection, CCR2KO mice were infected with *H. polygyrus*. Flow cytometry of blood confirmed that CCR2KO animals were deficient in Ly6Chigh (CCR2+; 16) monocytes compared to both naïve and infected WT animals (Fig. 4g; *p* < 0.005). According to published work (Epelman et al. 2014a), and our own data (Supplementary Fig. 1c) cMϕ may be divided into Ly6Chigh or Ly6Clow populations. Therefore, we subdivided the cMϕ to measure any effect CCR2 deficiency would have on these cMϕ populations. Total and Ly6Clow cMϕ number was higher in WT infected (black columns) vs naïve mice (open columns; Fig. 4h; *p* < 0.05, *p* < 0.01). There was no difference in cMϕ abundance between CCR2KO infected (grey columns) and naïve (grey and white striped columns) mice (Fig. 4h). cMϕ number was similar between naïve WT and CCR2KO animals (Fig. 4h).

## Discussion

This study has shown that cMϕ are not abundant or overtly polarised in healthy murine heart and their role her may be primarily phagocytic. However, the Mϕ of the heart react readily to Th2 challenge, adopting an ‘M2’ phenotype in response to helminth parasite infection. This expansion occurs primarily due to monocyte recruitment rather than resident cell proliferation in the heart, in contrast to some other tissues, demonstrating tissue specific variability in the capacity of resident cells to proliferate locally. Th2 challenge is also associated with increase in tissue fibrosis.

While some recent studies have reported a plentiful Mϕ population in the murine heart (Pinto et al. 2012; Heidt et al. 2014) this study, in agreement with others (Frangogiannis et al. 2002; Nahrendorf et al. 2007; McSweeney et al. 2010; Macri et al. 2012; Yan et al. 2013), describes the naïve mammalian heart as having a relatively sparse Mϕ population. Published studies report a Mϕ population in the heart of a few hundred/mg tissue (Molawi et al. 2014; Lavine et al. 2014) which is similar to our finding of around 250 Mϕ/mg. However, the absolute number of Mϕ present in the heart can change with age, with older animals (Macri et al. 2012) having a higher density of cMϕ. As well as this, diet, environment and sex may also affect on cMϕ number, as these factors are known to modify macrophage populations in different compartments (Weisberg et al. 2003; Stanton et al. 2011; Scotland et al. 2011). It is also possible that differences within methods for detecting cMϕ numbers might produce seemingly differing results.

This investigation also reveals that cMϕ are evenly distributed throughout the murine heart, which is in agreement with Azzawi et al. who describe human cMϕ as also being found throughout the myocardium (Azzawi et al. 1997). Interestingly this study and the present one also show that the distribution is more variable within the ventricles compared to the septum and that cMϕ can be associated with blood vessels, but are not exclusively so (Azzawi et al. 1997).

Macrophages isolated from the myocardium displayed a high capacity to phagocytose fluorescent microspheres. This is consistent with recent reports in which they have been found to phagocytose both apoptotic cardiomyocytes (Epelman et al. 2014a) and microbial pathogens (Heidt et al. 2014). A large proportion of cMϕ (∼50%) express the phagocytotic receptor CD206, also an ‘M2’ macrophage marker. These studies suggest that a crucial function of cMϕ in the steady state may be to scavenge cell debris/apoptotic cells in the heart in order to maintain homeostasis, without inducing inflammation inappropriately.

Mϕ are highly plastic (Mosser and Edwards 2008; Mylonas et al. 2009), and can adapt to stimuli in their environment. Opposing M1/M2-inducing signals can result in specific polarisation, or when present together can result in an intermediate phenotype (Yan et al. 2013; Mylonas et al. 2009; Vogel et al. 2013). The cMϕ population reported in the current study is not overtly polarised, expressing both ‘M2’ (Ym1, IL-10) and ‘M1’ markers (IL-1β and TNF) at low levels. Recent data has revealed that the cMϕ compartment progressively changes over time (Molawi et al. 2014), with cMϕ derived from embryonic progenitor cells gradually replaced by monocyte-derived Mϕ. Embryonic-derived Mϕ have been found to have greater reparative capacity than monocyte-derived, which are inherently more pro-inflammatory (Lavine et al. 2014). Therefore, depending on the age of the animal, the cMϕ population may have predominantly reparative (M2-like) properties, or appear more inflammatory (M1-like).

Helminth parasite infection induces a dramatic change in the characteristics of the cMϕ population. In *S. mansoni* infected mice the cMϕ population is expanded and becomes strongly ‘M2’-like, expressing increased amounts of Ym1 and RELMα compared to naïve mice. Ly6G immunohistochemistry of heart sections at this time reveals few neutrophils (data not shown), implying that these cells are not the source of Ym1. The *S. mansoni* infected mice also have a greater collagen content in the heart, increasing fibrosis. Similarly, the number of Mϕ and ‘M2’ Mϕ is significantly increased in the hearts of mice infected with another Th-2-inducing parasite *H. polygyrus*. Proliferation and alternative activation of peritoneal Mϕ has been reported previously in mice infected with the GI nematode (Jenkins et al. 2013). That we find only a trend in Ym1 increase and no RELMα in the heart with *H. polygyrus* could be due to the different lengths of infection time between the two parasites (8 weeks for *S. mansoni* vs 28 days for *H. polygyrus*). It may also be that *S. mansoni* generates a stronger, more systemic Th2 response than *H. polygyrus*.

One of the key cytokines responsible for polarisation towards a ‘M2’ phenotype is the Th2 cytokine, IL-4 (Gordon and Martinez 2010). Infections with helminth parasites, such as *S. mansoni*, induce large amounts of IL-4 systemically through the circulation (MacDonald et al. 2002). In helminth-associated Th2 cell settings, expansion of local Mϕ in serosal compartments (peritoneal and pleural cavities) can rely solely on proliferation of tissue-resident Mϕ exclusive of monocyte recruitment, a process predominantly under control of IL-4Rα signalling (the receptor for IL-4/IL-13) (Jenkins et al. 2011, 2013). However, this does not appear to be strictly the case in the heart, as most of the cMϕ expansion due to *H. polygyrus* infection was dependent on the recruitment of CCR2+ monocytes. This is perhaps unsurprising when one considers that cMϕ are replaced by blood monocytes during inflammation of the heart (Epelman et al. 2014a), and even during homoeostasis under normal conditions (Molawi et al. 2014). Despite this, our data shows no difference in cMϕ number in naïve WT vs CCR2KO mice suggesting that the cMϕ population can also maintain its numbers by *in situ* proliferation in the absence of monocytes. Even so, systemic Th2 cytokine production could underlie expansion and polarisation of the cardiac macrophage population in helminth infected mice, and this merits further investigation.

Clinical studies have investigated the effect of chronic helminth infection on cardiovascular disease, mostly in developing countries where parasite infections are relatively high, but atherosclerosis and cardiovascular event rates are low (Magen et al. 2005; Wiria et al. 2012). It has been postulated that the inverse relationship between helminth infection and cardiovascular events could be related to induction of a chronic Th2 activation state. In this state, upregulation of cytokines such as IL-4, IL-10 and IL-13 will modulate monocyte activation and downregulate disease associated inflammation e.g. in atherosclerotic plaques (Ritter and Moll 2000; Pinderski et al. 2002; Magen et al. 2005). To our knowledge, no work previously has examined the role of helminth infection directly on Mϕ in the heart. However, related to this, a recent study revealed that Th2 cytokines are important in the resolution of inflammation/injury in the heart, as after myocardial injury, survival and adequate healing was diminished in the absence of IL-13. This was associated with a decrease in markers of M2 activation in cMϕ (Hofmann et al. 2014). The present study would indicate that the anti-inflammatory, resolution-promoting (Th2) environment induced in the heart with helminth infection, which could have important implications for regulating the inflammatory response of the heart to subsequent injury. Whether this is ultimately beneficial leading to increased tissue repair after damage or detrimental due to increased fibrosis resulting in adverse remodelling in a highly Th2 environment, has yet to be determined.

## Conclusion

This study demonstrates the presence of a sparse resident, evenly distributed Mϕ population in the murine heart, which is not overtly polarised but has phagocytic capabilities, implicating a primarily scavenging role in the steady state to maintain homeostasis. However, the Mϕ population increases in number, largely through monocyte recruitment, and becomes stellate in shape and adopts an M2-like phenotype when infected with multicellular parasites. That infections remote to the site of the heart could be affecting the cMϕ may have important implications for their role in modulating the inflammatory response of the heart to subsequent cardiac events.

## Author contributions

GAG and KJM conceived, designed the experiments and wrote the manuscript. KJM carried out flow cytometry, FACS, realtime PCR, phagocytosis assay and histology. SJJ provided the *Cx3cr1*^*GFP/+*^ and CCR2KO mice and contributed to the manuscript. RFPC carried out FACS of liver and heart macrophages. KM carried out histology. ATP-A and ASM carried out *S. mansoni* infections, provided hearts and contributed to the manuscript. JPH, SMC and DR carried out *H. polygyrus* infections of mice, provided hearts and contributed to the manuscript. JEA provided *Cx3cr1*^*GFP/+*^ hearts and reviewed the manuscript.

## Conflict of interest

There are no conflicts of interest.
