# Supplementary material for: The adult murine heart has a sparse, phagocytically active macrophage population that expands through monocyte recruitment and adopts an ‘M2’ phenotype in response to Th2 immunologic challenge
Source: Immunobiology. 2015 Jul;220(7):924–33. doi: 10.1016/j.imbio.2015.01.013 (PMC4451497; doi:10.1016/j.imbio.2015.01.013)
Supplement: Supplementary file 1 [file mmc1.pptx]

## Slide 1
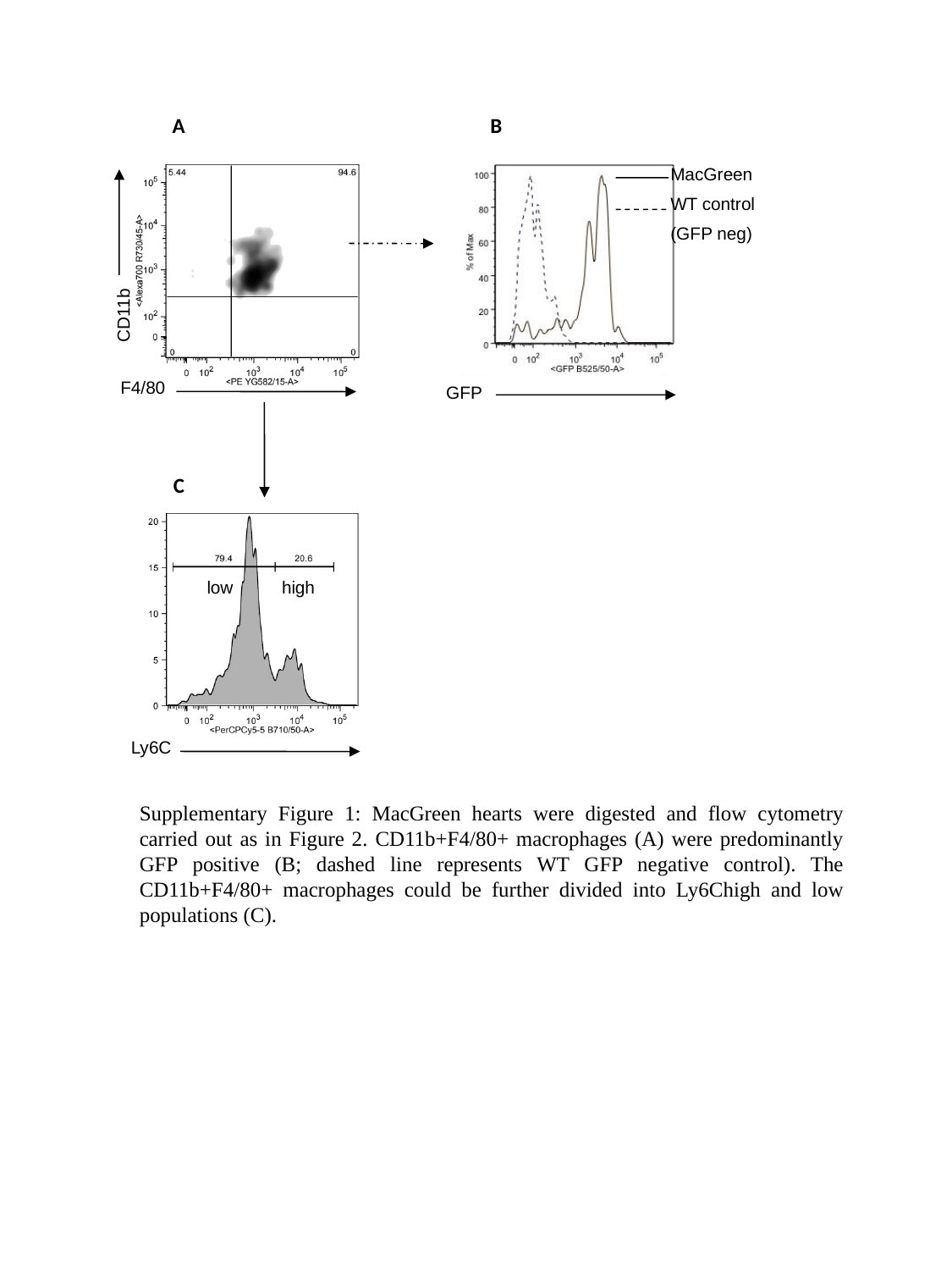

A
B
MacGreen
WT control
(GFP neg)
CD11b
F4/80
GFP
C
low high
Ly6C
Supplementary Figure 1: MacGreen hearts were digested and flow cytometry carried out as in Figure 2. CD11b+F4/80+ macrophages (A) were predominantly GFP positive (B; dashed line represents WT GFP negative control). The CD11b+F4/80+ macrophages could be further divided into Ly6Chigh and low populations (C).

## Slide 2
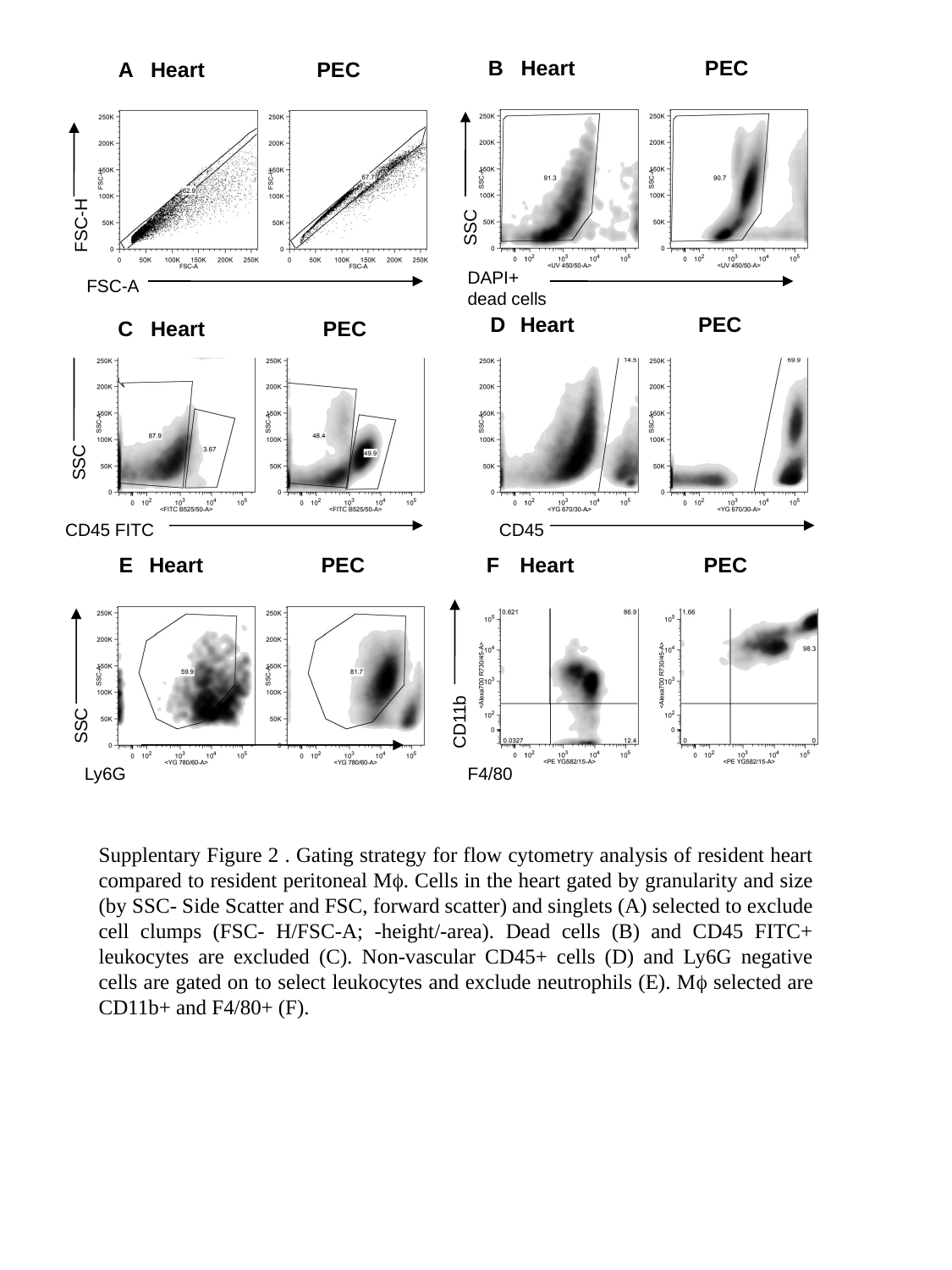

B Heart PEC
A Heart PEC
FSC-H
SSC
DAPI+
dead cells
FSC-A
D
Heart PEC
C Heart PEC
SSC
CD45 FITC
CD45
E
 Heart PEC
F
Heart PEC
CD11b
SSC
F4/80
Ly6G
Supplentary Figure 2 . Gating strategy for flow cytometry analysis of resident heart compared to resident peritoneal Mφ. Cells in the heart gated by granularity and size (by SSC- Side Scatter and FSC, forward scatter) and singlets (A) selected to exclude cell clumps (FSC- H/FSC-A; -height/-area). Dead cells (B) and CD45 FITC+ leukocytes are excluded (C). Non-vascular CD45+ cells (D) and Ly6G negative cells are gated on to select leukocytes and exclude neutrophils (E). Mφ selected are CD11b+ and F4/80+ (F).

## Slide 3
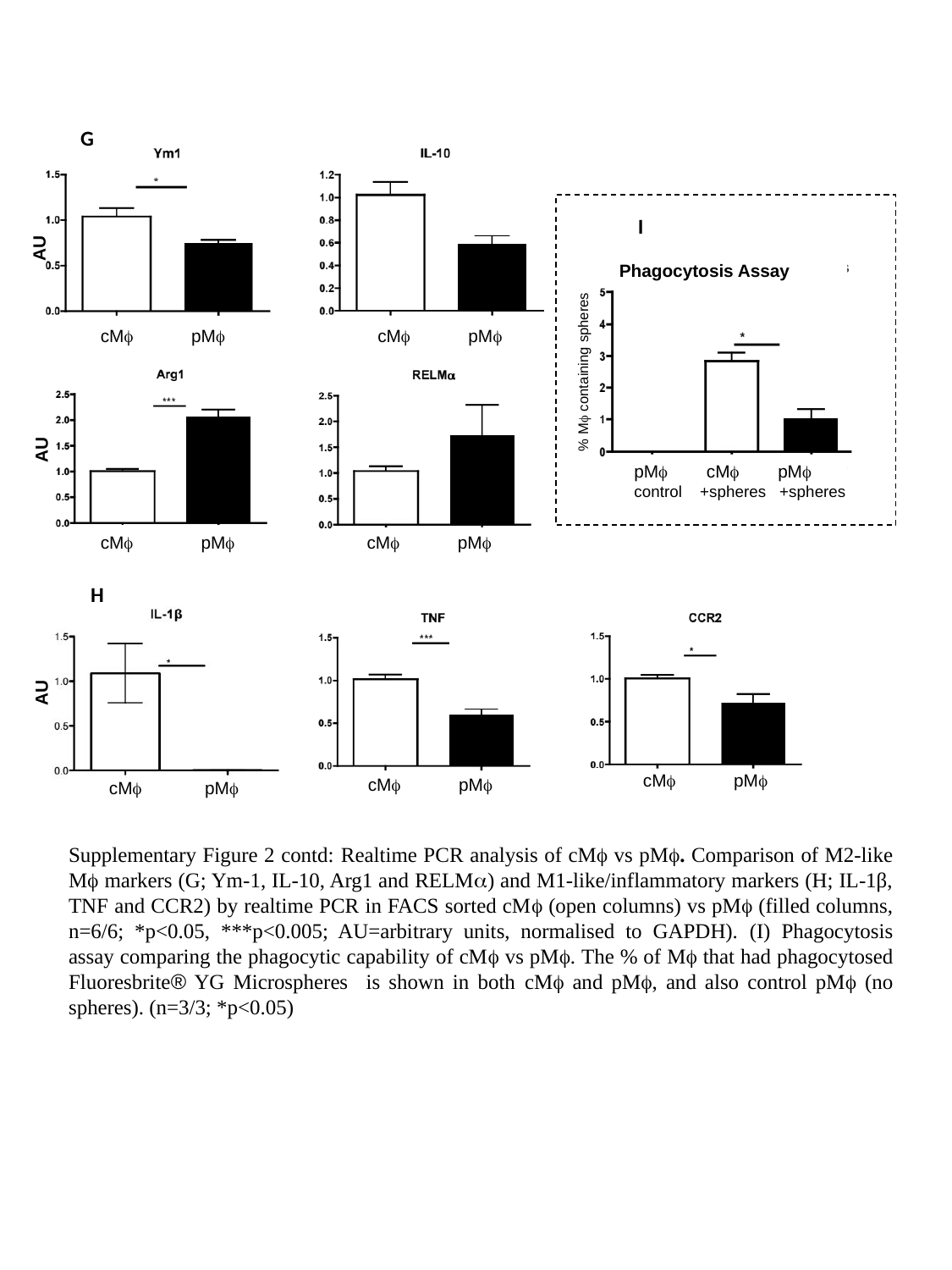

G
I
AU
Phagocytosis Assay
cMφ pMφ
cMφ pMφ
% Mφ containing spheres
AU
pMφ cMφ pMφ
control +spheres +spheres
cMφ pMφ
cMφ pMφ
H
AU
cMφ pMφ
 cMφ pMφ
cMφ pMφ
Supplementary Figure 2 contd: Realtime PCR analysis of cMφ vs pMφ. Comparison of M2-like Mφ markers (G; Ym-1, IL-10, Arg1 and RELMα) and M1-like/inflammatory markers (H; IL-1β, TNF and CCR2) by realtime PCR in FACS sorted cMφ (open columns) vs pMφ (filled columns, n=6/6; *p<0.05, ***p<0.005; AU=arbitrary units, normalised to GAPDH). (I) Phagocytosis assay comparing the phagocytic capability of cMφ vs pMφ. The % of Mφ that had phagocytosed Fluoresbrite® YG Microspheres is shown in both cMφ and pMφ, and also control pMφ (no spheres). (n=3/3; *p<0.05)
